# Can Robots Improve Testing Capacity for SARS-CoV-2?

**DOI:** 10.2196/20169

**Published:** 2020-08-12

**Authors:** Kathrin Cresswell, Sandeep Ramalingam, Aziz Sheikh

**Affiliations:** 1 The University of Edinburgh Edinburgh United Kingdom

**Keywords:** robotics, testing, SARS-CoV-2, COVID-19, pandemic, virus, infectious disease

## Abstract

There is currently increasing interest internationally in deploying robotic applications for severe acute respiratory syndrome coronavirus 2 (SARS-CoV-2) testing, as these can help to reduce the risk of transmission of the virus to health care staff and patients. We provide an overview of key recent developments in this area. We argue that, although there is some potential for deploying robots to help with SARS-CoV-2 testing, the potential of patient-facing applications is likely to be limited. This is due to the high costs associated with patient-facing functionality, and risks of potentially adverse impacts on health care staff work practices and patient interactions. In contrast, back-end laboratory-based robots dealing with sample extraction and amplification, that effectively integrate with established processes, software, and interfaces to process samples, are much more likely to result in safety and efficiency gains. Consideration should therefore be given to deploying these at scale.

## Introduction

Testing is crucial to identify, curb the spread of, and contain severe acute respiratory syndrome coronavirus 2 (SARS-CoV-2). Testing capacity will therefore need to increase substantially for the foreseeable future [1]. Robotic testing technologies may help to increase testing capacity and also minimize the risk of nosocomial transmission. There are currently two ways of testing for coronavirus disease (COVID-19): virological tests and serological tests. Virological methods work with genetic material obtained from nasal, throat, or saliva swabs and commonly use reverse transcription polymerase chain reaction (RT-PCR) technology to covert ribonucleic acid (RNA) to deoxyribonucleic acid (DNA). This technology is used to detect the presence of SARS-CoV-2. Serological tests use saliva, whole blood, serum, or plasma to look for antibodies. Various new testing methods that are variants of these two approaches are currently in development [2-6].

However, despite a general recognition that testing for SARS-CoV-2 is a key international priority, there is currently a lack of testing capacity contributing to the inadequate numbers of tests being undertaken [7]. In addition, existing testing procedures can endanger health care staff and laboratory technicians (ie, those who have to handle blood samples and swabs). We here provide an overview of key recent developments in robotic testing for SARS-CoV-2, which can help to reduce the exposure of health care and technical staff to the virus.

## Overview of Current Developments in Deploying Robots for SARS-CoV-2 Testing

**Figure 1:**
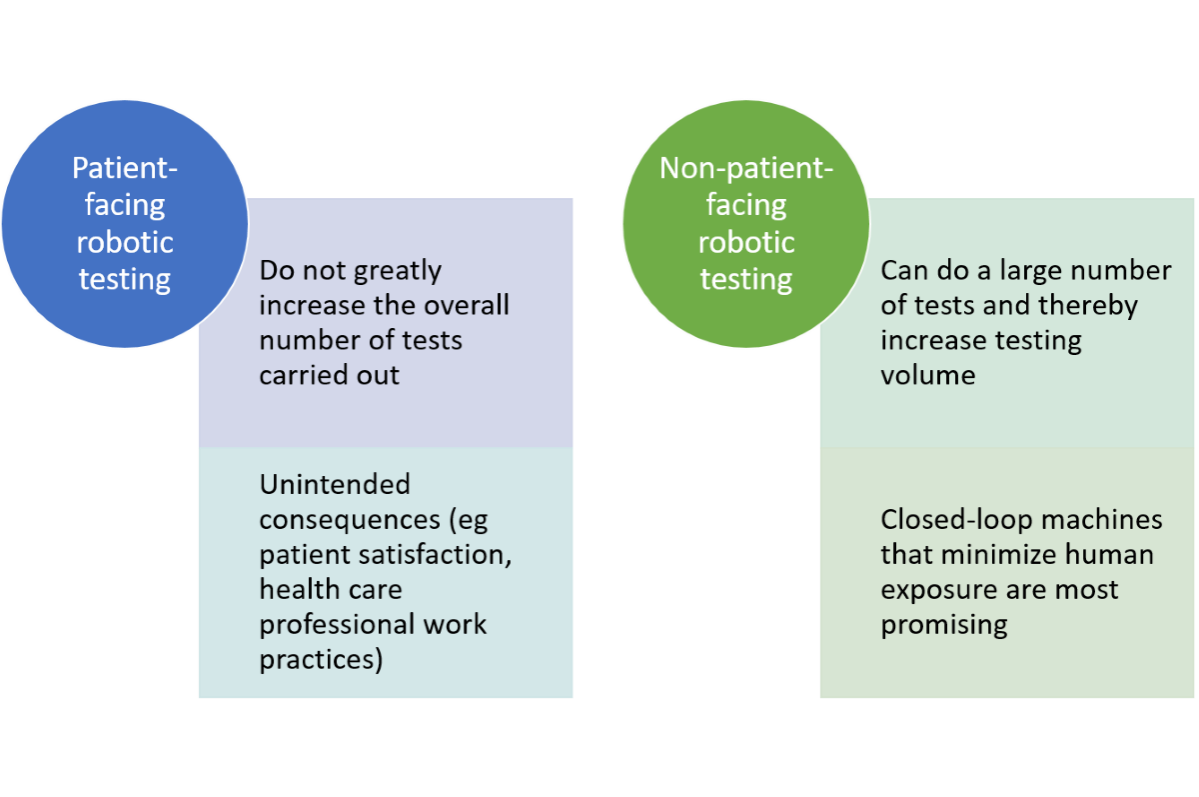
Types of robots used for severe acute respiratory syndrome coronavirus 2 (SARS-CoV-2) testing procedures.

Robots for SARS-CoV-2 testing procedures can be either patient-facing (eg, collecting nasal swabs and thereby reducing exposure of those collecting swabs) or non–patient-facing (eg, liquid handling machines that reduce exposure for laboratory technicians; Figure 1).

To date, patient-facing testing robots have been mainly experimental, deployed as pilots and in limited settings. Examples include remote-controlled robots taking throat swabs that have been used in parts of China [8], a robotic arm handing out test tubes to drivers in cars for SARS-CoV-2 testing [9], and a 3D-printed robotic arm taking throat swabs that was developed in Denmark [10]. Such robots are expensive (the Chinese robot costs approximately £62,000 [US $81,069]), can only do a limited number of activities on 1 sample at a time (and therefore do not greatly increase the overall number of tests carried out). They can also cause patients to worry due to a lack of personal contact [8]. Previous research has further found that patient-facing robotic applications can have unintended consequences resulting from adverse impacts on health care professional work practices, and on patient satisfaction [11,12]. For example, frail older adults and isolated patients depend on face-to-face contact as a source of emotional support. Attempts at making these applications more human-like may only partly address this issue, as robots that look too human-like can be perceived as threatening [13,14]. Patient-facing robots may, however, play a role in high-risk settings where infection control needs to be prioritized. They cannot replace face-to-face interactions that are required to provide high-quality and safe care for the majority of patients.

Non–patient-facing testing robots including liquid handlers, especially those that do not involve contact dispensing, are more promising. These robots can move liquids using magnetic plates, aspirate, dispense, or transport liquid samples (sometimes using pipettes), and in some cases interpret biological or chemical events (eg, detect if a virus is present). This reduces exposure for laboratory technicians who have to have to handle blood samples and swabs, and interface with these machines for sample preparation. Many laboratories already utilize some degree of automation, and this mitigates the risks of adverse impacts on existing work practices of health care staff.

Automated testing robots also have a high throughput and are rapidly able to tackle the large volumes of tests required during the SARS-CoV-2 pandemic and in the “new normal,” as they can carry out numerous tests simultaneously [15]. For example, the Spanish Ministry of Health recently commissioned 4 COVID-19 testing robots that will be able to carry out 80,000 tests per day [16]. Similarly, a newly established COVID-19 testing laboratory at Berkeley’s Innovative Genomics Institute (IGI) uses a robotic liquid handling machine that uses pipetting to test up to 3000 cases per day [17]. Another example is a Danish pipetting robot that automates the sample preparation process and was originally used for testing for *Salmonella* before being repurposed to test for SARS-CoV-2 [18].

However, the functionality of these machines varies. Some RT-PCR liquid handlers only help up to the extraction and addition of samples for PCR/RT-PCR, while others also transfer the material to a thermal cycler where PCR/RT-PCR happens (ie, extraction and amplification). Clearly, a closed-loop process (where technicians input a sample and the machine prepares samples and tests) is preferable as it minimizes human contact with samples (including testing for multiple viruses).

Another potential issue is the interfacing with existing software and the associated communication of results. Some automated testing robots do not allow automatic downloading of results from the robot to the main laboratory computer, which then renders the whole process impractical as the large number of results generated has to be manually entered by technicians. This may also introduce the risk of transcription errors, which may in turn have adverse consequences for patient care [19]. Additional integration software can help to address this issue, but adds to the overall cost (in relation to both acquisition and maintenance) and may require additional programming.

## Conclusions

Overall, there is a lack of empirical evidence on patient-facing virological/serological testing robots, and even if such evidence was available, these technologies would be unlikely to tackle pressing issues around scaling of testing capability. Testing robots in laboratories, however, have the potential to bring significant rapid benefits at low cost as these technologies can fulfil multiple purposes (eg, handling other types of liquids). Moreover, they already exist in many laboratories and can therefore be readily repurposed to respond to COVID-19 (although this has to be done by the manufacturer). Most useful are likely to be non–patient-facing testing robots that tackle the whole extraction and amplification cycle, as this will eliminate the need to transfer material for the amplification stage and thereby minimize the risk of unintended consequences.

Where there are established processes (eg, back-end laboratory-based robots tackling extraction and amplification) and where these interface effectively with existing software to process the results, these should be scaled up. In parallel, there is a need to stimulate research and innovation initiatives to explore the feasibility of developing a scalable front-end testing robot for high-risk settings (eg, infectious disease wards).

## Abbreviations

COVID-19: coronavirus disease
RT-PCR: reverse transcription polymerase chain reaction
SARS-CoV-2: severe acute respiratory syndrome coronavirus 2
